# Oxygen Pathway Modeling Estimates High Reactive Oxygen Species Production above the Highest Permanent Human Habitation

**DOI:** 10.1371/journal.pone.0111068

**Published:** 2014-11-06

**Authors:** Isaac Cano, Vitaly Selivanov, David Gomez-Cabrero, Jesper Tegnér, Josep Roca, Peter D. Wagner, Marta Cascante

**Affiliations:** 1 Center for respiratory diagnoses, Hospital Clinic and Institut d'Investigacions Biomèdiques August Pi i Sunyer (IDIBAPS) and Centro de Investigación Biomédica en Red en Enfermedades Respiratorias (CIBERES) and Universitat de Barcelona, Barcelona, Catalonia, Spain; 2 Departament de Bioquimica i Biologia Molecular, Facultat de Biologia, Universitat de Barcelona and Institute of Biomedicine (IBUB), Barcelona, Catalonia, Spain; 3 Unit of Computational Medicine of the Center for Molecular Medicine, Karolinska Institutet and Karoliska University Hospital - Department of Medicine, Stockholm, Sweden; 4 Division of Physiology, Pulmonary and Critical Care Medicine, University of California San Diego, San Diego, California, United States of America; Vanderbilt University Medical Center, United States of America

## Abstract

The production of reactive oxygen species (ROS) from the inner mitochondrial membrane is one of many fundamental processes governing the balance between health and disease. It is well known that ROS are necessary signaling molecules in gene expression, yet when expressed at high levels, ROS may cause oxidative stress and cell damage. Both hypoxia and hyperoxia may alter ROS production by changing mitochondrial *Po*_2_ (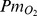). Because 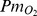 depends on the balance between O_2_ transport and utilization, we formulated an integrative mathematical model of O_2_ transport and utilization in skeletal muscle to predict conditions to cause abnormally high ROS generation. Simulations using data from healthy subjects during maximal exercise at sea level reveal little mitochondrial ROS production. However, altitude triggers high mitochondrial ROS production in muscle regions with high metabolic capacity but limited O_2_ delivery. This altitude roughly coincides with the highest location of permanent human habitation. Above 25,000 ft., more than 90% of exercising muscle is predicted to produce abnormally high levels of ROS, corresponding to the “death zone” in mountaineering.

## Introduction

It is well accepted that cellular hypoxia [1]–[4] triggers a constellation of biological responses involving transcriptional and post-transcriptional events [5], [6] through activation of cellular oxygen sensors. This includes generation of reactive oxygen species (ROS). Yet, current knowledge on the quantitative relationships between mitochondrial *Po*_2_ (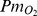), hypoxia-induced cellular events, and on the release of ROS from the inner mitochondrial membrane, is minimal. For example, how low 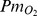 must be to trigger abnormally high ROS generation has not yet been identified. Furthermore, the levels of 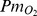 at rest and during exercise are unknown, although myoglobin-associated *Po*_2_ has been measured in intact exercising humans [7], [8], and is reported to be 3–4 mm Hg, implying that 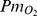 is likely even lower. There are promising new approaches being developed to in vivo assessment of 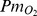 [9].

Recently, we extended a prior model describing O_2_ transport from the air to the mitochondria as an integrated system limiting maximal oxygen uptake 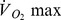 [10], [11] to also include the contribution to overall impedance to O_2_ flow from the above-zero 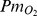 required to drive mitochondrial respiration [12]. This was accomplished by including an equation for the hyperbolic relationship between mitochondrial *Po*_2_ and mitochondrial 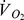 as shown by Wilson et al. [13] and confirmed more recently by Gnaiger's group [14], [15]. This has enabled the prediction of 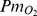 as a balance between the capacities for muscle O_2_ transport and utilization [12], [16]. In addition, we have expanded this model by now allowing for functional heterogeneity in both lungs and muscle [16], which had previously (and reasonably) been taken to be negligible in health. This was done to enable application to disease states. Since mitochondrial ROS generation is affected by cellular oxygenation, this integrative model may afford the opportunity for better understanding the quantitative relationship between O_2_ transport, mitochondrial respiration and ROS generation, provided we have an understanding of the relationship between 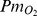 and ROS formation.

Recent modeling and experimental studies on mitochondrial ROS production under hypoxia and re-oxygenation [17]–[19] have proposed an inherent bi-stability of Complex III, i.e. coexistence of two different steady states at the same external conditions: one state corresponding to low ROS production, and a second potentially dangerous state with high ROS production. Temporary deprivation of oxygen could switch the system from low to high ROS production, thus explaining the damaging effects of hypoxia-re-oxygenation. This recently proposed model provides a conceptual basis for the abnormally high ROS production observed both in hyperoxia [20], [21] and hypoxia [1]–[4] and has the ability to predict the quantitative relationship between ROS generation and 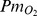.

In this paper, we build upon these previous but separate models of O_2_ transport as a physiological system and mitochondrial metabolism as a biochemical system, and have linked them through their common variables, 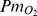 and 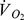, establishing one integrated model to predict 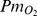 and mitochondrial ROS production. Specifically, we integrate the physiological model of the oxygen pathway predicting 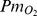 from the balance between O_2_ transport and mitochondrial O_2_ utilization [10]–[12], [16] with the model of electron and proton transport in the mitochondrial respiratory chain, and the ROS production associated with this transport [17]–[19], thereby predicting the rate of ROS production as a function of 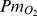 and 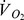. The former model is referred to below as the O_2_ pathway model, and the latter as the mitochondrial ROS prediction model.

In addition to describing the integrated modeling system, we present estimates of ROS generation in exercising muscle of healthy subjects at different altitudes at and above sea level using O_2_ transport data from Operation Everest II [22] and published mitochondrial kinetic data in normal human muscle (discussed in [17]). We found that at sea level, O_2_ transport at maximal exercise is sufficient to keep 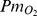 high enough that mitochondrial ROS generation is not significantly increased. However, exercise at high altitude is predicted to significantly increasing ROS generation, agreeing with experimental data collected under these conditions [23]–[25].

## Materials and Methods

### Oxygen pathway model

The modeling of O_2_ transport and utilization [10]–[12] adopted in the current article relies on the concept that maximal mitochondrial O_2_ availability is governed by the integrated behavior of all steps of the O_2_ transport and utilization system rather than being dominated by any one step. Building on the work of DeJours [26], and Weibel et al [27], it was previously shown [10], [11] how each step (ventilation, diffusion across the alveolar wall, circulation, diffusion from the muscle capillaries to the mitochondria, and finally, oxidative phosphorylation itself) contributes quantitatively to the limits to maximum O_2_ uptake (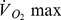). The model is based on the principle of mass conservation at every step, and uses the well-known mass conservation equations for each step as laid out by Weibel et al [28]. Given the oxygen transport properties of the lungs, heart, blood and muscles, and incorporating the (hyperbolic) mitochondrial respiration curve that relates mitochondrial 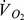 to mitochondrial *Po*_2_ [13]–[15] the model computes how much O_2_ can be supplied to the tissues (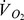), and the partial pressures of oxygen at each step (i.e. alveolar (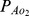), arterial (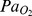), venous (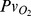), and mitochondrial (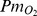)). This construct [12] leads to a system of five equations, each describing mass conservation equations governing O_2_ transport (Eqs. (1)–(4)) and utilization (Eq. (5)), with the five unknowns also mentioned above. 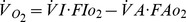
(1)

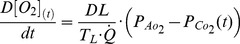
(2)

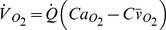
(3)

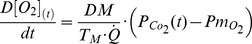
(4)

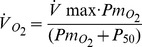
(5)


However, this system ignores heterogeneity of ventilation/perfusion (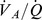) ratios in the lung, and heterogeneity of metabolism/perfusion (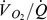) ratios in the muscle. This is laid out in detail in [16], and in the current article, consideration of typical, normal degrees of lung and muscle heterogeneity has been incorporated. The original 5-equation model [12] also failed to take into account that not all of the cardiac output flows to the exercising muscles. Allowance for blood flow to, and O_2_ utilization by, non-exercising tissues has accordingly also now been incorporated into the system described in [16] and is used here.

### Parameters of the O_2_ pathway model

The most complete data set with respect to O_2_ pathway conductances during exercise at altitude (ventilation (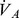), cardiac output (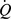), lung (*DL*) and muscle (*DM*) diffusional conductances, [Hb]) comes from Operation Everest II [22], but even here, measurements were made only at sea level, and at barometric pressures equivalent to four specific altitudes of 15,000 ft., 20,000 ft., 25,000 ft. and 29,000 ft. Because we wished to simulate the entire altitude domain from sea level to the Everest summit, we used the Operation Everest II data to interpolate parameter values at intervening altitudes. Additional input parameters are required for the analysis. These include: a) maximal muscle mitochondrial metabolic capacity (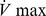), b) mitochondrial *P*_50_ i.e., the *Po*_2_ at which mitochondrial respiration is half-maximal, c) dispersion of ventilation/perfusion ratios in normal lungs, d) dispersion of metabolism/perfusion ratios in muscle, and e) an estimate of total blood flow to non-exercising tissues and their corresponding 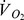.

For 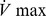, we chose a value 20% higher than the observed sea level maximal 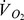 in the Operation Everest II subjects, and this came to 4.58 L/min. The justification is that in healthy fit subjects, 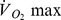 is O_2_ supply-limited at sea level, since it increases when 100% O_2_ is breathed [29], [30]. This demonstrates that mitochondrial metabolic capacity is clearly greater than sea level 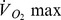. For mitochondrial *P*_50_, a value of 0.14 mm Hg was chosen. This is the mitochondrial *P*_50_ value used in the ROS prediction model described below [17]–[19], and is close to reported values measured in vitro [14], [15]. A representative normal level of ventilation/perfusion heterogeneity (i.e., 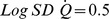) was used, since normal subjects display 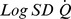 values of between 0.3 and 0.6 [31], increasing slightly with exercise. 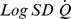 is the second moment (dispersion) of the perfusion distribution on a logarithmic scale and has been used to quantify 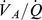 heterogeneity for about 40 years [32]–[34]. For 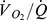 heterogeneity in muscle, there is very limited information. From a new technique based on near-infrared spectroscopy and currently in development, the corresponding dispersion in muscle appears to be about 0.1 (Vogiatzis et al, J. Applied Physiol, under revision), and this was used.

Finally, to allow for non-exercising tissue perfusion and metabolism we used typical normal resting values of cardiac output and 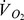 (specifically, non-exercising total tissue blood flow equal to 20% of maximal sea level cardiac output, and 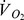 of 300 ml/min).

### Mitochondrial ROS prediction model

The mitochondrial ROS prediction model [17]–[19] considered in this study accounts for: i) Respiratory complex I that oxidizes NADH and reduces ubiquinone (Q), translocating H+; ii) Respiratory complex II that also reduces Q, oxidizing succinate to fumarate; iii) Respiratory complex III, the net outcome of which is to oxidize ubiquinol, reducing cytochrome c and translocating H+; iv) Respiratory complex IV that oxidizes cytochrome c, reducing molecular oxygen to H_2_O and translocating H+; and, v) The H+ gradient utilization for ATP synthesis in respiratory complex V. NADH consumed by complex I is produced in the several reactions of the TCA cycle leading from pyruvate to succinate, and from fumarate to oxaloacetate.

This model consists of a large system of ordinary differential equations that simulate the processes mentioned above based on the general principle of the law of mass action. Simulating the redox (from reduced/oxidized) reactions between the electron carriers constituting complexes I and III, the model takes into account that the carriers occupy fixed positions and have fixed interactions in the space of a respiratory complex. The variables of this model are the concentrations of redox states of the complex. Each redox state of the complex is a combination of redox states of the electron carriers constituting it. Since a carrier can be in one of the two redox states (reduced or oxidized), the number of variables is 2n, where n is the number of electron carriers fixed in the space of the complex. The model accounts also various forms of the complexes created by binding/dissociation of ubiquinone/ubiquinol. The other respiratory complexes are accounted for in a simplified form, assuming that the electrons that leave complex III ultimately reduce oxygen. Such a reaction is taken to be a hyperbolic function of oxygen concentration and proportional to the concentrations of forms able to donate an electron.

The mechanism of electron transport includes steps where highly active free radicals are formed. These radicals are capable of passing their unpaired high-energy electron directly to oxygen, thus producing superoxide anion radical and then the whole family of ROS such as OH radical and peroxides. The system of equations that constitutes this model is described in [17]–[19]. Parameter values for the respiratory chain used here were obtained from experimental studies [17] of normal mitochondrial function. The model calculates the levels of free radicals of the electron carriers constituting the respiratory chain (such as semiquinone radical). These radicals, normally formed in the process of electron transport, are responsible for ROS production. These levels are presented here as indicators of ROS production rate.

**Figure 1:**
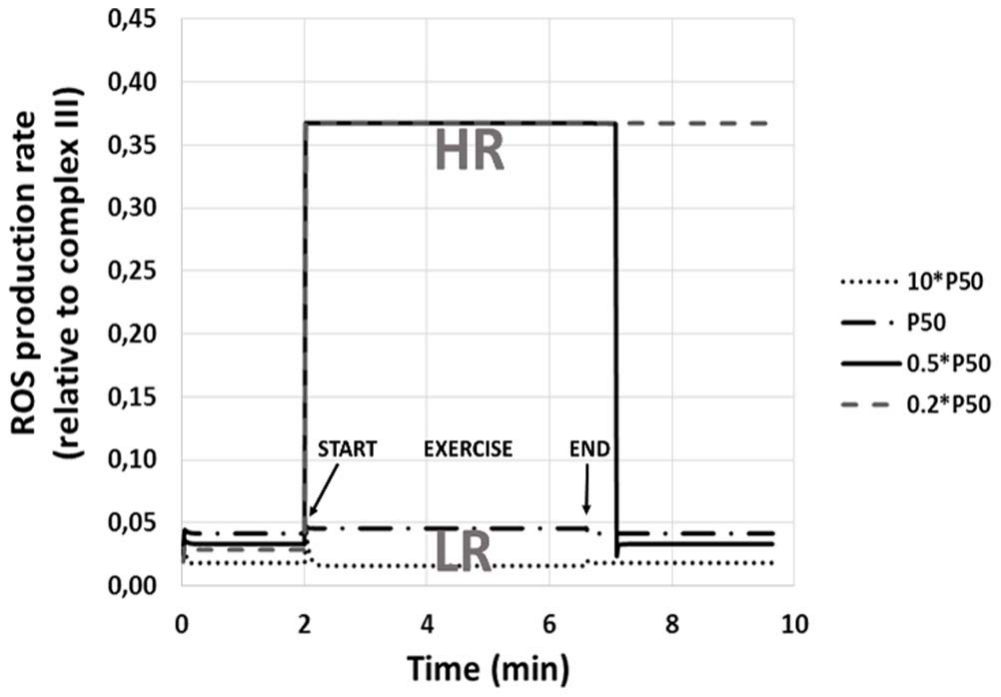
Dynamics of ROS production (expressed as SQo produced, normalized to total complex III abundance (taken as 0.4 nmol/mg mitochondrial protein)) at four steady state concentrations of oxygen (expressed as mitochondrial *Po*_2_ relative to *P*_50_, the oxygen partial pressure at the half-maximal rate of respiration). Before and after exercise, rest is simulated (no ATP hydrolysis, proton gradient dissipates only due to membrane leak). Between 2 and 6.6 min, exercise is simulated (membrane proton gradient dissipates due to ATP hydrolysis and ATP synthase activity). Overall, ROS production falls into two distinct patterns: one (HR, seen when 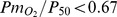) reflects high ROS generation and the other (LR, when 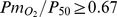) reflects little or no ROS generation compared to rest. Note post-exercise persistence of high ROS generation, especially at the lowest 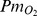 to *P*_50_ ratio.

Overall, the mitochondrial ROS generation model produces two distinct patterns of response to 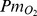 at maximum exercise, as displayed in **Figure 1**. One pattern (HR) reflects above normal high ROS generation and the other (LR) reflects little or normal ROS generation. Briefly, the figure shows the rate of mitochondrial ROS production expressed as concentration of semiquinone radicals (nmol/mg) at Qo site (ubiquinone binding site to complex III at the outer side of the inner mitochondrial membrane) of mitochondrial complex III (y-axis) against time (x-axis) for four different values of 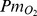, indicated in **Figure 1**.

Note that here, 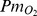 is expressed not as absolute values but as multiples of mitochondrial *P*_50_ (in this case from 0.2⋅*P*_50_ to 100⋅*P*_50_). For pattern LR, there is essentially no change in ROS generation, and this corresponds to the two 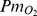 values exceeding the *P*_50_ in **Figure 1**. Pattern HR is seen in the two examples where 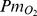 is less than *P*_50_, and here a large, almost 10-fold increase in ROS generation occurs. In fact, the switch occurs abruptly when 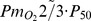. The high sensitivity of mitochondrial ROS production to restrictions of oxygen transport, and thus to low 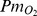, is a consequence of multistationarity, the mechanisms of which are considered in detail in previous publications [17], [18]. Finally, the increase in ROS generation persists after cessation of exercise, and the lower the 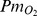, the longer the persistence time, also shown in **Figure 1**.

The non-linear bi-stability inherent to mitochondrial ROS prediction model [17]–[19] accounts for the abrupt change of mitochondrial ROS production rate with a change of external (with respect to the respiratory chain) conditions. In principle such a change can be irreversible (in accordance with the phenomenon of bistability investigated in [17]–[19]), but in the considered case it reverses with a delay. The delay results from slow oxidation of ubiquinol to ubiquinone, which is necessary to activate the Q-cycle in respiratory complex III.

### Procedure for model integration

**Figure 2:**
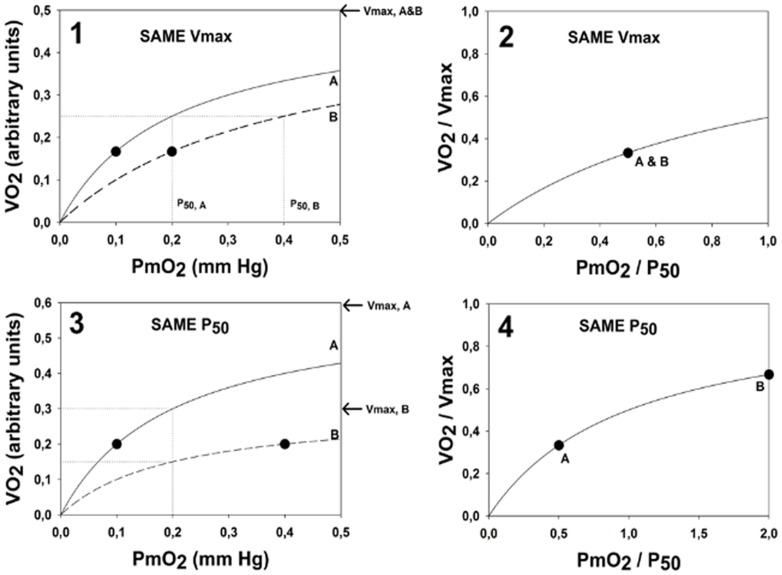
Importance of normalizing both actual 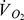 to mitochondrial 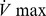 and actual mitochondrial *Po*_2_ (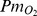) to mitochondrial *P*_50_ when 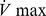 and/or *P*_50_ may vary within or between muscles. Panel 1: Example of two muscles (A & B) with the same 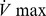 but different *P*_50_ that happen to have the same absolute 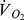 (closed circles). Although 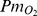 is lower in A than B, normalization of both axes (Panel 2) shows that in this case, 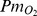 relative to *P*_50_ is the same, and this means that ROS generation will be the same for A and B. Panel 3: Example of two muscles (A & B) with the same *P*_50_ but different 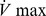 that again happen to have the same absolute 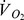 (closed circles). 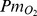 is again lower in A than B, but normalization of both axes (Panel 4) shows that 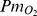 relative to *P*_50_ is lower in A than B, and this means that ROS generation will be high for A and normal for B.

The use of 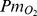 normalized to *P*_50_ reflects the fact that while the model predicts different ROS generation rates at different 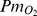 values for a given mitochondrial respiration curve of any particular *P*_50_, when 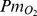 is the same fraction of *P*_50_, ROS generation is the same even if absolute 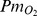 and *P*_50_ are different. For example, if 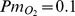 and *P*_50_ = 0.2, ROS generation would be the same as that computed when 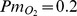 and *P*_50_ = 0.4, because in both cases, 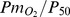 is the same ( = 0.5). This is illustrated in **Figure 2**, panels 1 and 2. In Panel 1, two mitochondrial respiration curves are drawn with the above different absolute *P*_50_ values (but the same 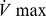 values). Using the above 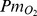 values, 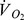 in both cases would be the same at 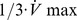, shown by the solid circles. Panel 2 replots these data normalizing the x-axis by *P*_50_ for each case, and normalizing the y-axis by 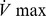 in each case.

These normalized respiration curves now overlie one another, and the important point is that the relative 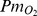 values in the two cases are identical and the relative 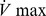 values are also identical, as will be ROS generation by the two regions. In a similar fashion, it is important to recognize that different muscle tissue regions may have different numbers of mitochondria and thus different 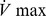 values even if *P*_50_ were uniform across regions. The normalization of 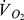 to 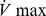 is thus necessary in order to compare different regions. For example, if 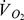 were the same at 0.2 units in two regions (and *P*_50_ were also the same in both regions) but 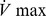
were different at 0.3 and 0.6 units in these two regions (**Figure 2**, panel 3), 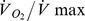
in the first region would be 0.67 but only 0.50 in the second region. When the data are replotted to normalize 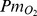 (x-axis) to *P*_50_ 0 and 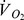 (y-axis) to 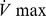 (**Figure 2**, panel 4), the two solid circles now separate. Thus, despite similar absolute 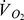 values, and the same *P*_50_, the second region lies lower on the mitochondrial respiration curve than the first region.

### Integration of the models

Recall that the oxygen pathway model consists of five equations, the solutions to which define the partial pressures of O_2_ at each step between the air and the mitochondria as well as the mass flow of O_2_ through the system (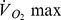). Key input variables for this model are mitochondrial 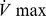 and 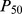 (along with all of the conductances of the O_2_ pathway). The key output variable for linkage to the metabolic model is 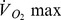 (as a fraction of 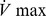). Thus, once the O_2_ pathway model has been run for any set of input variables, the metabolic model accepts as inputs from the O_2_ pathway model 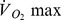, 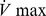, and mitochondrial *P*_50_, which also then defines mitochondrial *Po*_2_ from the hyperbolic mitochondrial respiration curve.

After scaling these variables to harmonize the units between the two models (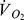 is in ml/min in the pathway model, but in nanomoles/min/mg mitochondrial protein in the metabolic model) the metabolic model is run, simulating mitochondrial respiration, i.e. the electron flow that reduces the transported oxygen to H_2_O. The principal outcome of the metabolic model for the present purposes is the rate of generation of ROS at exercise conditions for the given values of 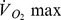, 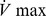, *P*_50_ and 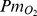.

## Results

**Figure 3:**
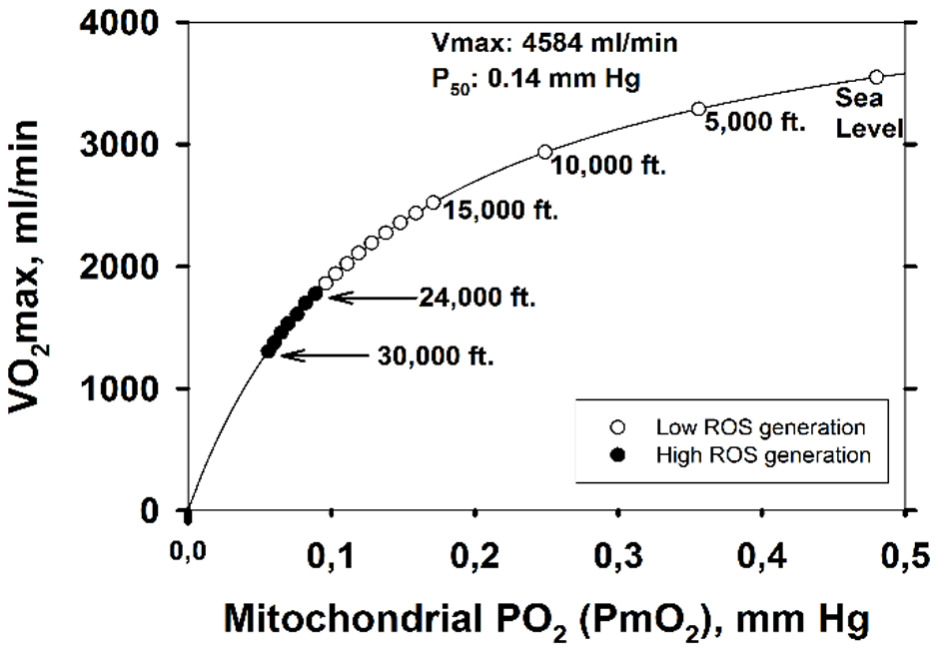
Reduction of maximal 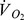 and mitochondrial *Po*_2_ in a *homogeneous* muscle as a function of altitude, (solid line, computed from the O_2_ pathway model) and degree of ROS generation as a function of mitochondrial *Po*_2_ (computed from the mitochondrial respiration model). Below 24,000 ft, ROS production is low (open circles), but above 24,000 ft, ROS production abruptly increases (closed circles).

The main outcome of this study is allowing a quantitative analysis of how the physiological O_2_ transport pathway [10]–[12] affects mitochondrial ROS generation in muscle [17]–[19]. **Figure 3** shows how maximal 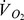 and mitochondrial *Po*_2_ in a homogeneous muscle will fall together with altitude, as computed from the O_2_ pathway model. **Figure 3** also displays the degree of ROS generation as a function of mitochondrial *Po*_2_ computed from the mitochondrial respiration model. As explained in [17]–[19], ROS generation abruptly switches from low to high levels when mitochondrial *Po*_2_ reaches a critical value of 2/3 of the *P*_50_ of the mitochondrial respiration curve – in **Figure 3** at about 0.1 mm Hg since *P*_50_ is 0.14 mm Hg. In this case, the corresponding altitude is 24,000 ft. above sea level. Thus, open circles (altitudes less than 24,000 ft.) reflect a state of low ROS generation, and closed circles (altitudes greater than 24,000 ft.) reflect a state of high ROS generation. While **Figure 3** shows the outcome for a homogeneous muscle, in reality the muscles will not be perfectly homogeneous, just as no random group of humans will all have exactly the same height or weight. The important type of heterogeneity for O_2_ transport in muscle is that of the ratio of mitochondrial metabolic capacity (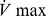, reflecting ability to consume O_2_) to blood flow (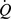, reflecting O_2_ availability). Thus, with heterogeneity, some muscle regions will have lower than average 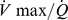 ratio, and other will have a 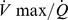 ratio greater than average. Data on the extent of heterogeneity in muscle are scarce due to lack of methods for its measurement, but recent, unpublished estimates based on near-infrared spectroscopy technology (Vogiatzis et al, J. Applied Physiol, under review) suggest that a small amount of heterogeneity does exist.

**Figure 4 pone-0111068-g004:**
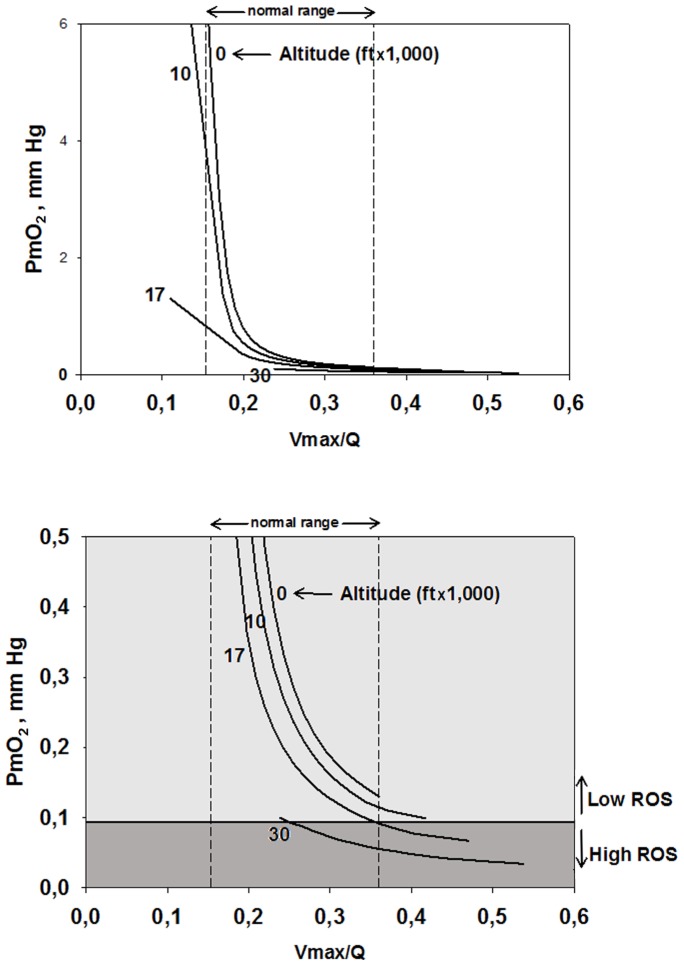
Mitochondrial *Po*_2_ (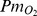) as a function of regional ratios of metabolic capacity (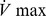) to blood flow (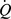) at four altitudes. The lower 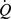 (supply) is in relation to 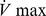 (demand), the lower is 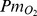 at any altitude; also, 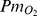 at any 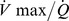 ratio falls with increasing altitude. Vertical dashed lines mark the normal range of 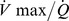. Both panels show the same data, but the lower panel expands the y-axis in its lower range to show when ROS generation is high (i.e., when 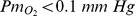). Below 17,000ft, ROS generation remains low, but above this altitude, regions of normal muscle with high 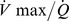 ratio generate high ROS levels, until at the Everest summit, almost the entire muscle generates high ROS levels.

When 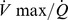 is high (metabolic capacity high in relation to O_2_ availability), mitochondrial *Po*_2_ will be low, and vice versa as shown in **Figure 4** (simulated for several altitudes from sea level to 30,000 ft.). When expressed as the second moment of the 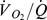 distribution, on a log scale, the value is about 0.1. This can be compared to the identically computed and well-established index of ventilation/perfusion (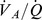) inequality in the normal lung of 0.3–0.6 [31], which is generally regarded as small. **Figure 4** also shows the range of 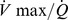 ratios for a muscle with normal heterogeneity (i.e., dispersion of 0.1) as from about 0.15 to about 0.36, pointing out the large range of mitochondrial *Po*_2_ that this seemingly small amount of heterogeneity creates. Thus, muscle regions with a high 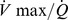 ratio become susceptible to high ROS generation before those with lower 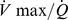 ratio. With the critical switch from low to high ROS production occurring at a 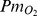 of about 0.1 mm Hg, **Figure 4** shows that with normal heterogeneity, the muscle regions with highest 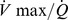 exhibit high ROS production already at 17,000 ft. altitude, and that at the summit of Mt. Everest (approx. 29,000 ft.), almost 100% of muscle regions will have switched to high ROS production.

**Figure 5:**
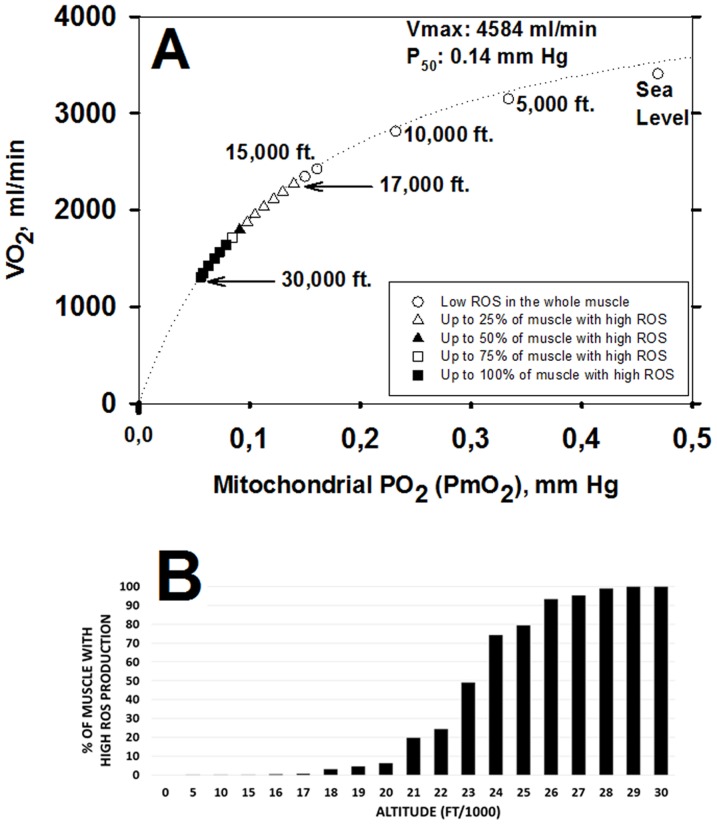
Effect of altitude on ROS generation when considering typical values for lung and muscle heterogeneities. A: Between 0 and 17,000 ft., ROS generation is within the normal range throughout the exercising muscle (open circles). Open triangles indicate that between 17,000 and 22,000 ft., abnormally high levels of ROS are predicted in up to 25% of exercising muscle (in regions with highest metabolic capacity in relation to O_2_ transport). The closed triangle (23,000 ft.) indicates high ROS in 25 to 50% of muscle. The open square (24,000 ft.) indicates 50–75% of muscle has high levels of ROS and filled squares (25,000–30,000 ft.) show that 75–100% of muscle regions express high levels of ROS (see text for more details). B: shows in more detail the percentage of exercising muscle that generates abnormally high levels of ROS at each altitude.

**Figure 5A** shows the consequences of normal muscle 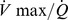 heterogeneity for the development of high ROS production in the format of **Figure 3**. The important points are: i) that due to the presence of high 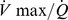 regions, high ROS generation is seen (in those regions) already at 17,000 ft., a much lower altitude than for the homogeneous system (24,000 ft.), and ii) that high ROS generation becomes more extensive with further increases in altitude. **Figure 5B** shows the percentage of muscle predicted to have high ROS production over the altitude range from sea level to the Everest summit.

## Discussion

The results displayed in **Figures 3** **–** [Fig pone-0111068-g004] **5** are specific to the input data used (Tables S1 and S2 in the supplementary on-line material). While they take advantage of the most complete data set available on humans exercising over a range in altitude from sea level to the equivalent of the Everest summit, the quantitative outcomes presented in this article would be different if a different data set were used. This should be kept in mind when interpreting the results presented. In addition, some specific, important data are both scarce in the literature and uncertain. The most important of these are the mitochondrial respiration curve characteristics (here defined by two parameters, 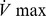 and *P*_50_), and the extent of heterogeneity in the distribution of blood flow to muscle regions with different metabolic capacity.

With respect to the mitochondrial respiration curve characteristics, in general, 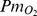 is systematically higher for the lowest 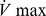, and at any 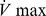, increasing mitochondrial *P*_50_ results in systematically higher 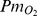 values. Because of uncertainty in 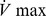 and *P*_50_ we carried out a sensitivity analysis that shows that in normoxia, as 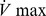 is varied from 10% to 20% to 30% above measured 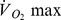, oxygen transport and utilization is unaffected at 3.8 L/min. However, 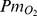 varies somewhat (1.2, 0.7, 0.5 mm Hg). At altitude, the effects were similar: at 15,000 ft. oxygen consumption was invariant at 2.8 L/min and 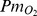 was 1.9, 1.6, 1.3 mm Hg respectively; while at 30,000 ft. oxygen consumption remained invariant at 1.4 L/min with 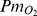 values of 0.07, 0.06, 0.06 mm Hg respectively. At any altitude, therefore, prediction of ROS generation was not affected by this degree of uncertainty in 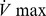.

When *P*_50_ was varied between 0.1 and 1.0 mm Hg, oxygen utilization was minimally affected (3.8 to 3.6 L/min respectively) while 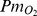 increased from 0.5 to 3.7 mm Hg. Considered relative to *P*_50_, which is what is important in ROS generation as discussed above, 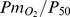 decreased from 4.9 to 3.7. At 15,000 ft. oxygen utilization fell from 2.8 to 2.7 L/min as *P*_50_ was raised from 0.1 to 1 mm Hg and 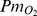 increased from 0.2 to 1.5 mm Hg. When normalized to *P*_50_, 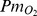 was invariant at 1.5⋅*P*_50_. At 30,000 ft. oxygen utilization was unaffected as *P*_50_ was varied from 0.1 to 1 mm Hg (at 1.4 L/min), while 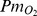 increased from 0.05 to 0.45 mm Hg. However, when normalized to *P*_50_, 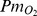 was invariant at 0.45⋅*P*_50_. Thus, variation in *P*_50_ over a 10-fold range did not affect the outcome in terms of ROS generation.

While the particular results we report depend on the values we took for these functions, the important point is the presentation of the integrated model approach coupling physiological elements of O_2_ transport with biochemical elements of oxidative phosphorylation stands, no matter what specific data are used to run it. A graphical user interface to parameterize and simulate the integrated model is freely available at https://sourceforge.net/projects/o2ros.

### Biological and clinical implications

It is of interest that our results suggest that ROS generation in exercising normal muscle switches to high levels already at 17,000 ft., or about 5,000 m. This is the altitude above which permanent human habitation does not occur [35], and also the altitude above which humans experience inexorable loss of body mass. It is easy to hypothesize a cause and effect relationship between ROS and these findings, given the generally pro-inflammatory effects of high ROS levels, but whether this is indeed cause and effect or just coincidence remains to be established. In the same vein, the widespread presence of high ROS generation within muscle above 20,000 ft. and almost uniform presence above 25,000 ft. coincides with what is popularly termed the “death zone” in the mountaineering community – altitudes where fatalities are common. Of course, bitter cold, high winds, and hypoxia itself are likely contributors to the high risk of death under these conditions, but it is possible that high ROS production may be playing a role, not just in muscle but perhaps also in critical organs such as the brain. There is a growing body of literature [36]–[38] suggesting that endogenous ROS at high concentrations are damaging to cells, while at lower levels they are involved in activating important signaling pathways, some of which relate to adaptation to hypoxia (angiogenesis, for example where the promoter region of the critical angiogenic gene VEGF has a binding site for H_2_O_2_ [39]. They also act as pro-survival molecules regulating kinase-driven pathways [36], [40]. A recent systems analysis of abnormal muscle bioenergetics in patients with Chronic Obstructive Pulmonary Disease (COPD) [41] provides indirect evidence for a central role of cellular hypoxia in explaining abnormal regulation of key metabolic networks regulated by genetic and epigenetic mechanisms. Moreover, there is evidence [42]–[44] of the role of nitroso-redox disequilibrium explaining systemic effects in several chronic disorders such as COPD, chronic heart failure and type II diabetes. However, current knowledge of mitochondrial dysfunction [45]–[47] is still incomplete. The centrality of oxygen metabolism in organisms leads to the notion that it is also involved in other complex chronic diseases at essentially every level of organization [5], [48]–[50].

Potential applications of the integrated transport/metabolic model, which has been presented here only in terms of healthy humans at altitude, are envisioned in systems medicine with analysis of the potentially greater degree of ROS generation associated with impaired O_2_ availability in patients with diseases such as COPD, heart failure, diabetes and peripheral vascular disease. With the necessary input data, this could be done for individual patients to assess the likelihood of high ROS generation in muscle. In addition, the model may allow the prediction of the benefits of exercise programs and pharmacological interventions in these patients. Through its predictions, the current analysis may open new avenues in assessment of the impact of impaired oxygen exchange on increased mitochondrial ROS generation as well as in evaluation of the consequences of oxidative stress on biological functions in different acute and chronic diseases [5], [6]. While prediction of mitochondrial ROS generation may be possible, it is clear that the current integrated model addresses only the relationships between determinants of cellular oxygenation and mitochondrial ROS production. It does not attempt to deal with other sources of ROS [51] or complexities of the redox system such as the antioxidant resources that will modulate the biological activity of ROS generated by exercise within muscle, and which will thus affect oxidative stress. Accounting for such elements is well beyond the scope of this article, but it's a target for the future.

## Conclusions

The integration of the two deterministic modeling approaches considered in this study allows us to establish a quantitative analysis of the relationships between the components of the O_2_ pathway [10]–[12], [16] and mitochondrial ROS generation [17]–[19]. To this end, the simulations herein using data from exercising normal subjects at sea level and altitude have shown that when 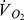 is low (less than 40% of mitochondrial oxidative capacity) due to impaired O_2_ transport, extremely low 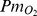 values will develop, which in turn may be associated with above normal ROS production. This will occur when 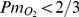 of mitochondrial *P*_50_. The current investigation may open new avenues for assessing the impact of impaired oxygen transport on increased mitochondrial ROS generation as well as for evaluating the consequences of oxidative stress on biological functions in both acute and in chronic diseases [5], [6].

## Supporting Information

Table S1 Input parameters for the oxygen transport system. Values interpolated from those measured during OEII (reference at sea level, 15,000 ft, 20,000 ft, 25,000 ft and 29,000 ft).
(DOCX)

Table S2 Input parameters for the modeling of Cell Bioenergetics and ROS production.
(DOCX)
