# Supplementary material for: Oxygen Pathway Modeling Estimates High Reactive Oxygen Species Production above the Highest Permanent Human Habitation
Source: PLoS One. 2014 Nov 6;9(11):e111068. doi: 10.1371/journal.pone.0111068 (PMC4222897; doi:10.1371/journal.pone.0111068)
Supplement: Table S1 — Input parameters for the oxygen transport system. Values interpolated from those measured during OEII (reference at sea level, 15,000 ft, 20,000 ft, 25,000 ft and 29,000 ft). (DOCX) [file pone.0111068.s001.docx]

**TABLE S1 – Input parameters for the oxygen transport system. Values interpolated from those measured during OEII (reference at sea level, 15,000ft, 20,000ft, 25,000ft and 29,000ft)**

| ***Altitude (ft.)*** | ***Barometric pressure (***$\boldsymbol{PB}$***), Torr*** | ***Body temperature (***$\boldsymbol{T}$***), °C*** | ***Hemoglobin concentration (***$\left[ \boldsymbol{Hb} \right]$***), g·dl^-1^*** | ***Alveolar ventilation (***$\dot{\boldsymbol{V}}$***), BTPS, L·min^-1^*** | ***Blood flow (***$\dot{\boldsymbol{Q}}$***), L·min^-1^*** | ***Total Lung O_2_ diffusing capacity (***$\boldsymbol{DL}$***), ml·min^-1^·Torr^-1^*** | ***Total muscle O_2_ diffusing capacity (***$\boldsymbol{DM}$***), ml·min^-1^·Torr^-1^*** |
| --- | --- | --- | --- | --- | --- | --- | --- |
| ***0*** | 760 | 38 | 14,2 | 128,3 | 25,0 | 50,9 | 104,6 |
| ***5000*** | 639 | 38 | 14,9 | 137,1 | 23,5 | 60,9 | 97,1 |
| ***10000*** | 534 | 38 | 15,5 | 145,9 | 22,0 | 70,9 | 89,6 |
| ***15000*** | 442 | 38 | 16,2 | 154,6 | 20,5 | 80,9 | 82,1 |
| ***16000*** | 426 | 37,9 | 16,3 | 156,4 | 20,2 | 82,9 | 80,6 |
| ***17000*** | 410 | 37,8 | 16,5 | 158,1 | 19,9 | 84,9 | 79,1 |
| ***18000*** | 394 | 37,7 | 16,6 | 159,9 | 19,6 | 86,9 | 77,6 |
| ***19000*** | 380 | 37,6 | 16,7 | 161,6 | 19,3 | 88,9 | 76,1 |
| ***20000*** | 365 | 37,5 | 16,9 | 163,4 | 19,0 | 90,9 | 74,6 |
| ***21000*** | 351 | 37,4 | 17,0 | 165,2 | 18,7 | 92,9 | 73,1 |
| ***22000*** | 337 | 37,3 | 17,1 | 166,9 | 18,4 | 94,9 | 71,6 |
| ***23000*** | 324 | 37,2 | 17,2 | 168,7 | 18,1 | 96,9 | 70,1 |
| ***24000*** | 311 | 37,1 | 17,4 | 170,4 | 17,8 | 98,9 | 68,6 |
| ***25000*** | 299 | 37 | 17,5 | 172,2 | 17,5 | 100,9 | 67,1 |
| ***26000*** | 286 | 37 | 17,6 | 173,9 | 17,2 | 102,9 | 65,6 |
| ***27000*** | 275 | 37 | 17,8 | 175,7 | 16,9 | 104,9 | 64,1 |
| ***28000*** | 264 | 37 | 17,9 | 177,4 | 16,6 | 106,9 | 62,6 |
| ***29000*** | 253 | 37 | 18,0 | 179,2 | 16,3 | 108,9 | 61,1 |
| ***30000*** | 243 | 37 | 18,2 | 181,0 | 16,0 | 110,9 | 59,6 |
