# Supplementary material for: Oxygen Pathway Modeling Estimates High Reactive Oxygen Species Production above the Highest Permanent Human Habitation
Source: PLoS One. 2014 Nov 6;9(11):e111068. doi: 10.1371/journal.pone.0111068 (PMC4222897; doi:10.1371/journal.pone.0111068)
Supplement: Table S2 — Input parameters for the modeling of Cell Bioenergetics and ROS production. (DOCX) [file pone.0111068.s002.docx]

**TABLE S2 – Input parameters for the modeling of Cell Bioenergetics and ROS production**
